# Editorial: Heterologous Protein Expression and Production Platforms: The How, Now and Wow of It, Volume II

**DOI:** 10.3389/fbioe.2022.946381

**Published:** 2022-07-13

**Authors:** Jacinta S. D’Souza, Siddhesh B. Ghag, Dong-Yup Lee

**Affiliations:** ^1^ School of Biological Sciences, UM-DAE Center for Excellence in Basic Sciences, University of Mumbai, Kalina Campus, Mumbai, India; ^2^ School of Chemical Engineering, Sungkyunkwan University, Suwon, SouthKorea

**Keywords:** recombinant proteins, genetic engineering, overexpression platform, expression tags, microbial factories

The process of protein production or expression in cells involves synthesis, modification and regulation. Since the emergence of genetic engineering tools and recombinant DNA technology, concerted efforts have been invested by basic scientists and application specialists to optimise procedures for recombinant protein production in the 1980s. The era began with an apt overexpression platform for a host, viz. *Escherichia coli*, and expanded alternative host systems, such as *Mycobacterium*, some *Bacillus* strains, *Caulobacter crescentus*, viruses, yeasts (*Hansenula*, *Kluyveromyces*, *Pichia*, *Saccharomyces* and *Yarrowia*), filamentous fungi (*Aspergillus*), algae (*Chlamydomonas reinhardtii*, *Schizochytrium* sp., *Synechococcus*), plants (*Arabidopsis*, tobacco), diatom *Phaeodactylum tricornutum*, or insect and mammalian cell cultures. The technology has certainly come of age with optimised strains, manipulatable genetic toolkits, expression of both intracellular as well as secretory proteins, and their ability to carry out post-translational modifications. This has resulted in its rampant use, both for basic and application-oriented fields and in the biopharmaceuticals with easily scalable protein expression platforms. All these systems offer their pros and cons and one is yet on the lookout for an overexpression platform that harbours the advantages of all these systems (in particular, a green platform).

We geared ourselves towards this research topic as it is of interest to a broad category of Scientists. Original research and review articles were invited and this is a sequel to Volume I which had a total submission of 9 out of which 6 were accepted. Volume II (current) had 11 submissions, out of which 6 were accepted. The current Editorial summarises accepted submissions of Volume II.

Mayrhofer et al. in the research titled, “**Functional Trimeric SARS-CoV-2 Envelope Protein Expressed in Stable CHO Cells**” have timely studied one of the most important viruses that have caused havoc all over the world in recent times, the severe acute respiratory syndrome coronavirus 2 (SARS-CoV-2). The group has provided a robust and easy method for recombinant expression and purification of the Spike protein. They have developed a recombinant CHO cell line that stably expresses the extracellular domain of the trimeric variant of the SARS-CoV-2 spike protein. Such a system would be useful to study ACE-2 interaction with the spike protein, important in the entry of the virus into the host cell. At a time when we still have an impending danger of the SARS-CoV-2 infecting humans, this platform will serve as very useful for basic and applied research in the field.

The study titled, **“Strategies to enhance periplasmic recombinant protein production yields in *Escherichia coli*”** by De Gier and Karyolaimos has systematically reviewed the field of producing recombinant proteins in the periplasm of *E. coli*. They discussed the overall benefits of using this expression platform, these being, disulphide bond formation is enabled, protein is isolated and hence not vulnerable to cleavage by cytoplasmic proteases, and control of the nature of the N-terminus of the mature protein. Nevertheless, problems that could affect periplasmic protein production also have been pointed out, these being disruption in protein targeting, protein instability, protein translocation and improper folding. To circumvent these pitfalls, the authors have discussed strategies that have been used to bring *in sync* two aspects of this system, namely, the capacity of the cell’s secretory apparatus with that of the rate of producing secretory periplasmic recombinant protein. Since the cell’s secretory apparatus is elaborate, it involves tuning the transcriptional and translational processes and selection of appropriate signal peptide. All this, to increase the efficiency of translocation and targeting, better folding capacity, adaptation of the host to the system under consideration.

The original research by Sathesh-Prabu et al. titled, “**A levulinic acid-inducible and tunable gene expression system for *Methylorubrum extorquens***” describes a low-cost inducible promoter for the tunable expression of genes of interest in *Methylorubrum extorquens*. The authors tested glucose-, xylose-, or levulinic acid (LA)-inducible promoters for the induction of reporter gene, *i.e.* green fluorescent protein (GFP). Among those listed above, the LA-inducible system with HpdR regulator and its cognate promoter PHpdH showed a stronger expression of GFP protein upon induction compared to the control. This system was able to induce GFP at a concentration of about 10% of the total protein and the expression was found to be dose-dependent. This inducible gene expression system would be beneficial to large-scale fermentation using *M. extorquens* in formate and methanol-based C1-bioeconomy.

Hwang et al. in the work titled, “**Streptomyces as microbial chassis for heterologous recombinant protein expression**” have reviewed the use of *Streptomyces* species for heterologous expression of biosynthetic gene clusters (BGCs) and recombinant proteins. Engineering *Streptomyces* for yield improvement is practiced through the development of genetic tools, chassis construction, and synthetic biology approaches. Further the authors also discussed the strategies to improve yield by exploiting strong promoters, signal sequences and secretory pathways. They also provided perspectives on the development of “specialized *Streptomyces* chassis library” by employing the design-build-test-learn cycle in systems.

Tan et al. in the study titled, “**HEK293 Cell Line as a Platform to Produce Recombinant Proteins and Viral Vectors**” have reviewed the use of human-based HEK293 cells and its sub-types as expression systems for the production of proteins and viral vectors. They argued in favour of having a ‘human-like’ post-translational system for producing biotherapeutics that are meant for use on humans. A summary of the advantages of using HEK293 over other cells makes this review comprehensive and an up-to-date summary of biotherapeutics and viral vectors produced thus far.

Plavec et al. in the study titled, “**Introduction of Modified BglBrick System in *Lactococcus lactis* for Straightforward Assembly of Multiple Gene Cassettes**” have developed a modified BglBrick system in lactic acid bacterium *Lactococcus lactis* by assembling multiple gene cassettes in a single pNBBX plasmid. This modified BglBrick system was used to drive the expression of six gene cassettes consisting of the promoter, gene for model proteins, and terminator sequence. The model proteins used were either protein binders that were expressed and subsequently displayed on the surface of the bacteria which were detected using flow cytometry or fluorescent proteins that were expressed intracellularly and determined by fluorescence measurements. Genetically engineered *L. lactis* finds application in industry and therapy where concomitant expression of multiple proteins is required. This study provides a proof of principle of expressing multiple proteins simultaneously in *L. lactis* bacteria.

We believe this Research Topic broadly covers relevant and interesting works toward characterising, designing and producing recombinant proteins in various expression hosts. Given that several researchers using recombinant technology to address questions in Biology, this Research Topic is timely and encourages Volume III.

## References

[B1] Hwang S. Lee Y. Kim J. H. Kim G. Kim H. Kim W. (2021). Streptomyces as Microbial Chassis for Heterologous Recombinant Protein Expression. Front. Bioeng. Biotechnol. 9, 804295. 34993191 10.3389/fbioe.2021.804295 PMC8724576

[B2] Karyolaimos A. de Gier J. W. (2021). Strategies to Enhance Periplasmic Recombinant Protein Production Yields in *Escherichia coli* . Front. Bioeng. Biotechnol. 9, 797334. 34970535 10.3389/fbioe.2021.797334 PMC8712718

[B3] Mayrhofer P. Hunjadi M. Kunert R. (2021). Functional Trimeric SARS-CoV-2 Envelope Protein Expressed in Stable CHO cells. Front. Bioeng. Biotechnol. 9, 779359. 34976974 10.3389/fbioe.2021.779359 PMC8718689

[B4] Plavec T. V. Ključevšek T. Berlec A. (2021). Introduction of Modified BglBrick System in Lactococcus lactis for Straightforward Assembly of Multiple Gene Cassettes. Front. Bioeng. Biotechnol. 9, 797521. 34957084 10.3389/fbioe.2021.797521 PMC8703077

[B5] Sathesh-Prabu C. Ryu Y. S. Lee S. K. (2021). Levulinic Acid-Inducible and Tunable Gene Expression System for *Methylorubrum extorquens* . Front. Bioeng. Biotechnol. 9, 797020. 34976985 10.3389/fbioe.2021.797020 PMC8714952

[B6] Tan E. Chin C. S. H. Lim Z. F. S. Ng S. K (2021). HEK293 Cell Line as a Platform to Produce Recombinant Proteins and Viral Vectors. Front. Bioeng. Biotechnol. 9, 796991. 34966729 10.3389/fbioe.2021.796991 PMC8711270

